# Ophthalmic findings in Cohen syndrome patient without subjective ophthalmic complaints: A case report

**DOI:** 10.1097/MD.0000000000035945

**Published:** 2023-11-17

**Authors:** Do Ah Kim, Joonhyung Kim

**Affiliations:** a Department of Ophthalmology, CHA Bundang Medical Center, CHA University, Seongnam, Republic of Korea.

**Keywords:** Cohen syndrome, genetic disorder, hereditary retinal disease

## Abstract

**Rationale:**

Cohen syndrome is a rare genetic disorder that can cause various symptoms, including ophthalmic manifestations that can significantly impact a patient’s visual health and quality of life.

**Patient concerns:**

We present the case of a 12-year-old boy diagnosed with Cohen syndrome who exhibited retinal degeneration and macular edema but could not express ophthalmic symptoms due to a developmental disability.

**Diagnoses:**

The patient was diagnosed with Cohen syndrome by a heterozygous mutation in the *VPS13B* gene by whole exome sequencing and referred to ophthalmology for an ophthalmic examination.

**Intervention:**

Ophthalmologic tests, including visual acuity, intraocular pressure, slit lamp examination, fundus photography, and optical coherence tomography, were performed.

**Outcomes:**

Visual acuity and intraocular pressure were not measured due to poor cooperation, and no abnormal findings were observed on the slit lamp examination. However, peripheral retinal degeneration was observed in the fundus examination, and cystoid macular edema was observed in both eyes on optical coherence tomography.

**Lessons:**

Regular ophthalmologic examination is important for patients with Cohen syndrome, especially those with developmental disabilities who may not be able to express their symptoms. Clinicians should be aware of the potential ophthalmologic manifestations of Cohen syndrome and the importance of timely diagnosis and management.

## 1. Introduction

Cohen syndrome is a rare autosomal recessive disorder characterized by various clinical features, including obesity, hypotonia, mental deficiency, and craniofacial, ocular, and limb anomalies. It was first reported in 1973 by Cohen and his colleagues.^[1,2]^ The prevalence of Cohen syndrome is not known, but it is estimated to be 500 to 1000 cases worldwide.^[3]^ The diagnosis of Cohen syndrome is made based on clinical features and can be confirmed with genetic testing, identifying pathogenic variants in VPS13B.^[4]^ Ophthalmic manifestations are common in patients with Cohen syndrome and can significantly impact visual health and quality of life.^[5]^ The aim of this report is to present the ocular features of a 12-year-old boy diagnosed with Cohen syndrome, who exhibited retinal degeneration and macular edema, but was unable to express ophthalmic symptoms due to a developmental disability.

## 2. Case report

**Figure 1.**
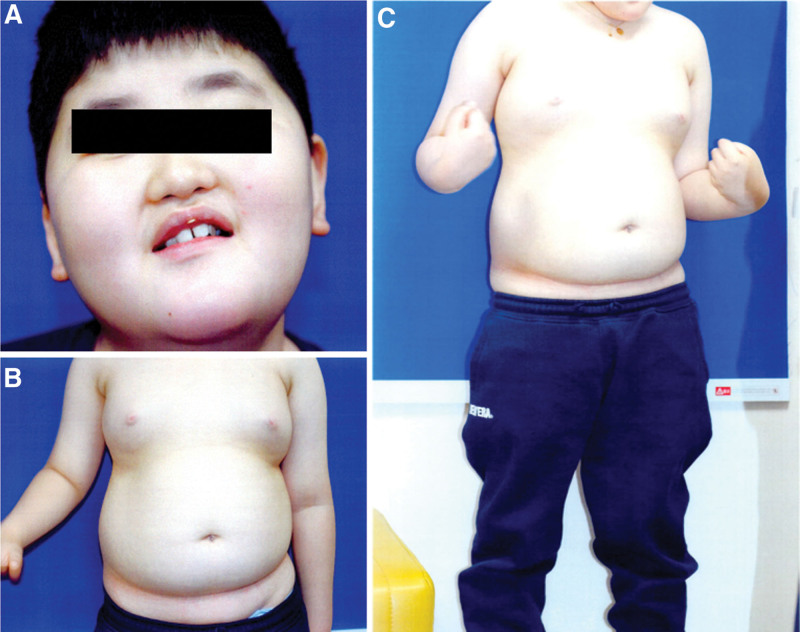
External photo of the patient. (A) Facial gestalt. Short and upturned philtrum, beak shaped nose, and open-mouthed expression. (B) Truncal obesity. (C) Tapering fingers.

**Figure 2.**
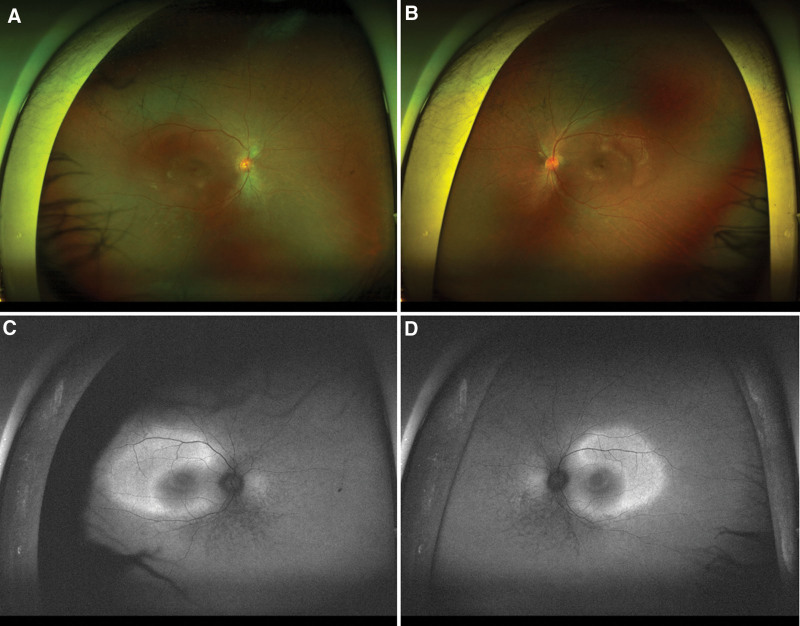
Fundus photographs of the patient. Chorioretinal degeneration in both eyes.

**Figure 3.**
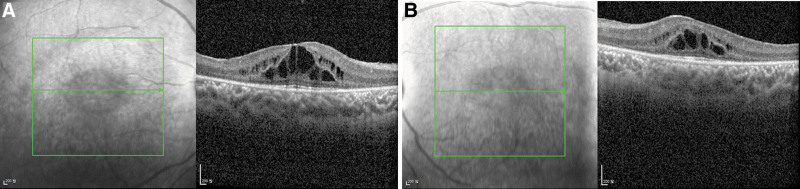
Optical coherence tomography of the patient. (A) Retinoschisis-like changes with cystoid macular edema in right eye. (B) Cystoid macular edema in left eye.

A 12-year-old boy, diagnosed with Cohen syndrome by a heterozygous mutation in the *VPS13B* gene, was referred to ophthalmology for an ophthalmic examination. He exhibited a shortened and upturned philtrum, prominent upper incisors, and abdominal obesity (Fig. 1). Due to a developmental disability, he was unable to express ophthalmic symptoms. According to the parents, there were no apparent ophthalmologic discomforts in the patient. Visual acuity and intraocular pressure were not measured due to poor cooperation, and no abnormal findings were observed on slit-lamp examination. The axial length was 21.41 mm in the right eye and 21.44 mm in the left eye. Refraction was +3.00 diopters sphere in the right eye and +2.25 diopters sphere in the left eye. Fundus examination revealed peripheral retinal degeneration (Fig. 2). Macular optical coherence tomography revealed cystoid macular edema in both eyes (Fig. 3). A short-term follow-up was decided without treatment, and intraocular injection or oral medication will be considered if the macular edema worsens at the next visit.

For publication of this case report, written informed consent was obtained from the patient’s father.

## 3. Discussion

The main cause of vision loss in Cohen syndrome has been proposed to be high myopia and chorioretinal dystrophy. Myopia is mainly characterized by a refractive error resulting from dysgenesis and atrophy of the cornea, ciliary body, and iris, which in turn cause iridial and zonular laxity and spherophakia. The severity of myopia in Cohen syndrome is typically measured as median refraction of −8 diopters (range −0.25 to −18.0), and the progression of myopia begins around the age of 5 years. Unlike common myopia, the depth of the anterior chamber is shallow, which increases the risk of angle-closure glaucoma.^[6–8]^ The average axial length of a 12-year-old Korean children was 23.05 ± 0.58 mm. However, in our case, unlike myopia seen in Cohen syndrome,^[9]^ the axial length was shorter than that of the same age group and hyperopia was confirmed. Due to the patient’s developmental disorder, the visual acuity could not be measured as there was a lack of verbal expression.

Norio et al^[10]^ were the first to describe retinal changes, such as chorioretinal dystrophy with a bull’s-eye-like macula and pigmentary deposits, as well as optic atrophy in patients with Cohen syndrome. Taban et al reported that retinal dystrophy was recorded in 38% of patients with Cohen syndrome. Pigmentary retinopathy was seen in 91% of Finnish patients, retinal involvement in 83% of Japanese patients, and 32% of other Caucasians.^[11]^ Gabrielle et al^[12]^ reported that cystoid maculopathy is a frequent finding in Cohen syndrome, with a prevalence of about 80%. Nasser et al^[13]^ described bull’s-eye maculopathy, cystic changes, and atrophy of outer retinal layers in 5 patients, while Beck et al^[14]^ described a patient with non-leaking cystoid macular edema on fluorescein angiography.

The rate of progression of retinal diseases varies, but it generally progresses very slowly and continues to worsen with advancing age. The earliest fundus changes are a pale disc and a pale fundus, sometimes with pigment granularity. Over time, these changes are followed by narrowed vessels, pigment clumps, and bone spicule-like pigment accumulations, typically between the ages of 10 to 20. By 35 to 40 years of age, pigment deposits increase and approach the posterior pole. Patients over the age of 45 commonly experience severe chorioretinal atrophy, while a bull’s-eye macula is observed in most patients.^[7]^

In Korea, about 100 cases of Cohen syndrome have been reported so far,^[15]^ and in 2006, Park et al reported the first ophthalmic abnormality of Cohen syndrome. Fundus examination of a 13-year-old girl and a 14-year-old boy, both siblings, showed chorioretinal atrophy with bony pigmentation in both eyes. In our case, as in the previously reported case, bilateral cystoid macular edema and chorioretinal degeneration were observed.^[16]^

Vision loss in Cohen syndrome usually begins around the age of 5, and between the ages of 5 and 30, visual acuity is typically maintained between 0.5 and 1.0. However, as the patient ages beyond 30 years, visual acuity tends to deteriorate to Hand Motion or Light perception, although complete blindness does not typically occur.^[7]^ In the second decade of life, progressive constriction of visual fields is often observed, which can lead to significant impairment of visual function. Despite this, useful vision is usually preserved until the fourth decade.^[11]^

Early correction of visual defects, such as glasses to correct refractive errors or strabismus, has a positive effect on development.^[6,7,17]^ However, there is currently no effective treatment available to halt the progression of pigmentary retinopathy. Despite the relatively high rate of Cohen syndrome-associated retinopathy, there is still a lack of description and long-term longitudinal follow-up data on the management of this macular complication. Gabrielle et al reported that topical and systemic carbonic anhydrase IV inhibitors were administered for the treatment of cystoid maculopathy but failed to reduce or prevent the disease progression.^[12]^

Many patients with Cohen syndrome cannot speak complete sentences by age 6, have some degree of intellectual disability, and up to 22% show significant delays.^[18]^ In our case, the patient was unable to express himself and interact with others due to an underlying disorder affecting his language development. Although the patient did not report any discomfort, ophthalmologic examinations were performed with recognizing the ophthalmic abnormalities associated with Cohen syndrome, and these abnormalities were confirmed.

Early detection and management of ophthalmic abnormalities in patients with Cohen syndrome can help to prevent vision loss and improve the overall quality of life. Treatment options may include corrective lenses, low-vision aids, or surgical interventions, depending on the severity and nature of the ophthalmic abnormality. Regular ophthalmologic examinations can also help to monitor the progression of any existing abnormalities and detect new ones as they arise.

## 4. Conclusions

Our case report emphasizes the significance of routine ophthalmologic screening in patients diagnosed with Cohen syndrome, especially those with developmental disabilities who may not be able to express their symptoms. Clinicians must recognize the possibility of ophthalmic abnormalities in Cohen syndrome and promptly manage them to ensure the best possible visual outcomes. Patients with newly diagnosed Cohen syndrome should receive ophthalmic screening, and those with existing diagnoses should receive regular ophthalmic examinations. By doing so, we can effectively detect and manage ophthalmic problems in patients with Cohen syndrome, thus improving their overall quality of life.

## Author contributions

**Conceptualization:** Joonhyung Kim, Do Ah Kim.

**Supervision:** Joonhyung Kim.

**Writing—review & editing:** Joonhyung Kim, Do Ah Kim.

**Writing—original draft:** Do Ah Kim.
